# Preclinical screening for cardiovascular disease with high-sensitivity cardiac troponins: ready, set, go?

**DOI:** 10.3389/fcvm.2024.1350573

**Published:** 2024-11-14

**Authors:** Aya Awwad, Yashika Parashar, Soahum Bagchi, Saman Asad Siddiqui, Ogheneochuko Ajari, Christopher deFilippi

**Affiliations:** ^1^ Postgraduate Research Program, Harvard Medical School, Boston, MA, United States; ^2^ Inova Schar Heart and Vascular, Inova Health System, Falls Church, VA, United States

**Keywords:** troponin, biomarker, screening, risk, prevention

## Abstract

Cardiovascular disease (CVD), including atherosclerosis, valvular etiologies, or myocardial disorders, is typically asymptomatic for several years, representing an occult phase of illness. Readily available preventive treatments to reduce cholesterol and blood pressure, among other risk factors, have the potential to reduce and delay incident myocardial infarction (MI), heart failure, and cardiovascular (CV) deaths. Measurement of circulating levels of cardiac troponin T (cTnT) and troponin I (cTnI) released from cardiomyocytes, as a result of injury, has been the biochemical standard for the diagnosis of MI for more than 20 years. The recent adoption of high-sensitivity (hs) assays, which are capable of measuring cTnT and I levels in more than 50% of the general population, has revealed a clear association between progressively higher biomarker levels and future CV events. In cross-sectional imaging studies, cTn levels measured by hs assays have also demonstrated correlations between elevated biomarker levels and occult CVD such as coronary artery disease and myocardial fibrosis. In this review, we provide evidence to consider measuring hs-cTnT and hs-cTnI to screen for patient CV risk and provide an example of a scenario in which such screening may improve outcomes through decision support for aggressive management of blood pressure.

## 1 Introduction

Screening allows clinicians to detect disease in a preclinical phase, offering an opportunity to interrupt the natural progression to symptoms, morbidity, and, ultimately, premature mortality. Critical to the success of a screening program is not only the early detection of disease but also the demonstration of a resulting intervention that can delay or prevent further development of the disease process.

For cardiovascular disease (CVD), substantial effort has been expended to develop primary prevention algorithms to delay and prevent atherosclerotic CVD (ASCVD). ASCVD adverse outcomes are typically classified as coronary heart disease deaths, non-fatal MIs, and non-fatal and fatal strokes (1). The development and application of the 10-year pooled cohort risk calculator has been codified into the American Heart Association/American College of Cardiology (AHA/ACC) 2018 guidelines (1).

Substantial resources have been devoted to screening for early ASCVD and modifying ASCVD risk factors such as hyperlipidemia, but less attention has been directed toward the prevention of heart failure (HF). Annually in the United States, HF is associated with approximately 1,000,000 new diagnoses, 1.3 million hospitalizations, nearly 100,000 deaths, and expenses estimated at over $30 billion, making it one of the most costly medical conditions in the country (2). HF symptoms are often considered the result of multiple heterogeneous disorders, and unlike ASCVD, challenges have existed to classify a preclinical phase of the disease. These barriers have rendered it difficult to develop screening strategies.

The introduction of high-sensitivity cardiac troponin (hs-cTn) assays has revolutionized the clinical utility of cTns, moving beyond their traditional role as a dichotomous marker for acute myocardial infarction (MI). hs-cTn assays allow for the detection of very low concentrations of cardiac troponins, even in patients without overt MI. This capability has expanded their use to quantify cardiac myocyte injury and myocardial stress, providing more nuanced insights into cardiovascular risk. More recently, detectable levels of hs-cTnT or hs-cTnI have been observed in asymptomatic individuals and in patients with stable coronary artery disease (CAD), which is supposed to reflect ongoing subclinical myocardial damage.

In 2021, a universal definition for HF was developed that redefined stage B, the preclinical phase of the disease (3). The diagnosis of stage B HF relies on imaging evidence of cardiac pathology or notable elevation of circulating cardiac-specific biomarkers of cardiac stress and/or myocyte injury [natriuretic peptides (NPs) or cTnT or cTnI] (3). Measurement of these cardiac-specific biomarkers may offer a pragmatic component of early identification of individuals at higher cardiovascular risk during the preclinical phase of HF.

Progressively higher levels of both NPs and cTns are strongly associated with incident symptomatic HF, typically requiring hospitalization within the ensuing 5–10 years across multiple longitudinal cohorts of community-dwelling individuals without HF symptoms or known CVD (4, 5). While measurement of either NPs or cTns (measured with an hs assay) appears to be associated with a similar prospective risk of incident HF, the cost of measuring hs-cTns is less than a third of the cost of measuring NPs ($12.47 vs. $39.26 US dollars, Centers for Medicare and Medicaid services reimbursement Q3, 2024). Thus, measurement of cTns may represent a more attractive candidate for implementation in a large-scale, community-based screening program (6).

While there may be significant public health policy interest in defining preclinical disease using hs-cTn assays, widespread adoption of a clinical screening program will become possible only if a cost-effective and safe intervention is readily available.

## 2 The making of a screening biomarker

The process of evaluating a new biomarker typically consists of two critical steps: (1) analytical validation and (2) qualification (7). These steps are specific to each condition of use for the biomarker. Analytical validation refers to the assessment of available evidence on the analytical performance of an assay, while biomarker qualification represents the evidence-based process of linking a biomarker with one or more clinical endpoints (7). The analytical validation of hs-cTn assays has been previously described, as the biomarker is already in clinical use for diagnostic purposes. In the following sections, we will detail the existing evidence of observational data linking cTn with several clinical endpoints outside the diagnosis of MI. We will also review retrospective and *post-hoc* analyses of clinical trials that illustrate the effects of interventions on both the biomarker and clinical outcomes.

### 2.1 Emerging role of a single cardiac troponin measure in screening and risk stratification

**Figure 1:**
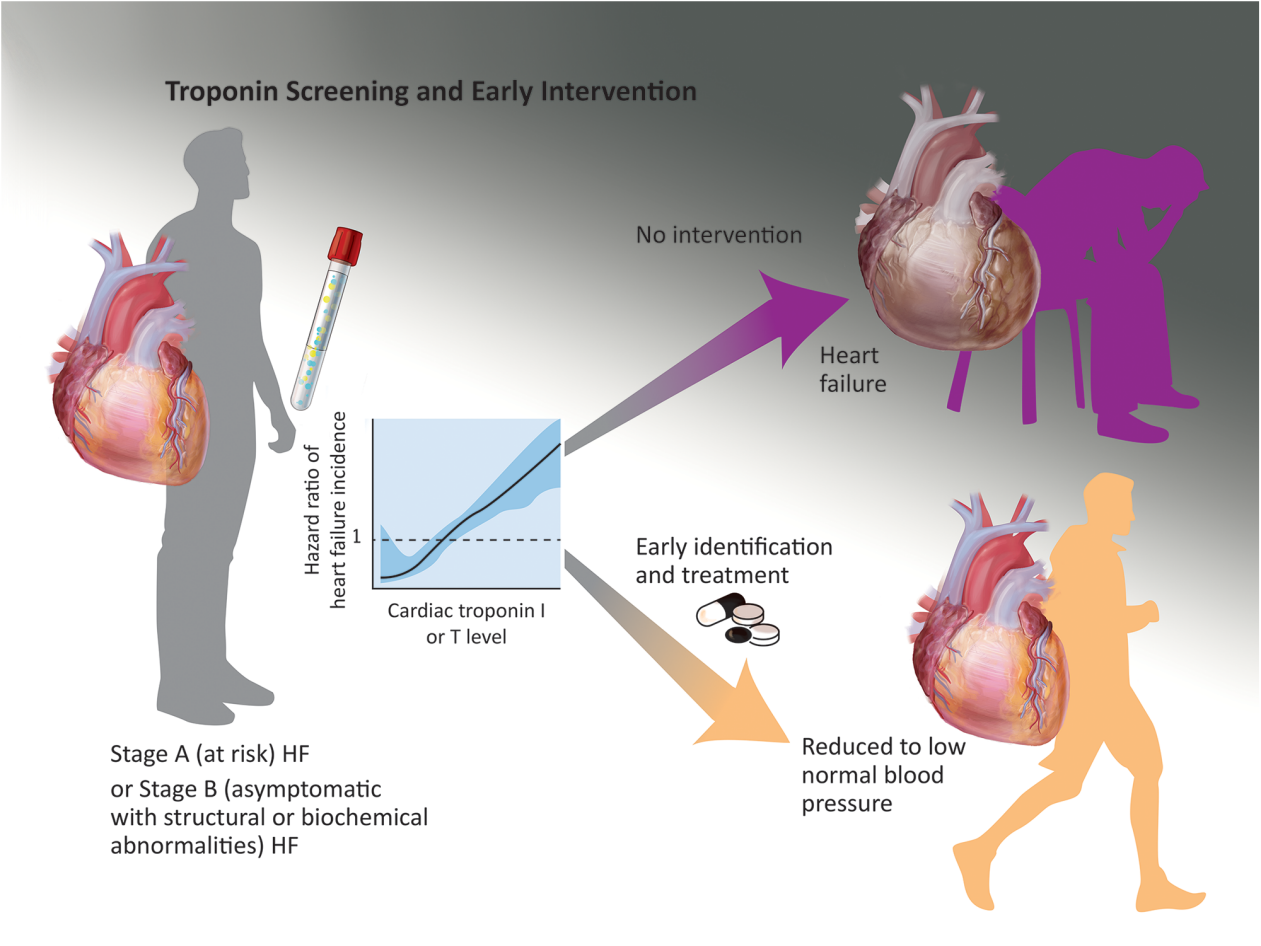
A potential screening strategy is identification of ambulatory individuals with stage A or B heart failure (risk factors or asymptomatic structural findings such as left ventricular hypertrophy) with early stages of hypertension; when these individuals are identified with elevated hs-cTnI or T levels and treated to low systolic blood pressure targets, they may experience a lower incidence of symptomatic heart failure and other adverse cardiovascular events in the next 5–10 years. This scenario requires prospective validation.

Driven by the analytical progress of hs-cTn assays that were originally designed for achieving superior early detection of MI compared to prior less-sensitive versions of the assays, there was recognition that circulating cTns levels are also measurable in the majority of community-dwelling adults. Notably, it has been demonstrated that elevated cTns levels below the diagnostic threshold of MI are associated with an increased number of CV events. As a result, there is growing interest in repurposing hs-cTn measurement for incident CVD and HF risk screening in the general population. Multiple studies have highlighted that individuals from ambulatory populations with elevated levels of cTns die early from CV and experience more adverse CVD outcomes, including HF, when compared with those with lower levels while adjusting for conventional CV risk factors. These findings have been further underscored by meta-analyses, collectively emphasizing the predictive efficacy of baseline hs-cTn measurements for long-term CV prognoses in ambulatory adults (Figure 1) (5, 8–11).

**Figure 2:**
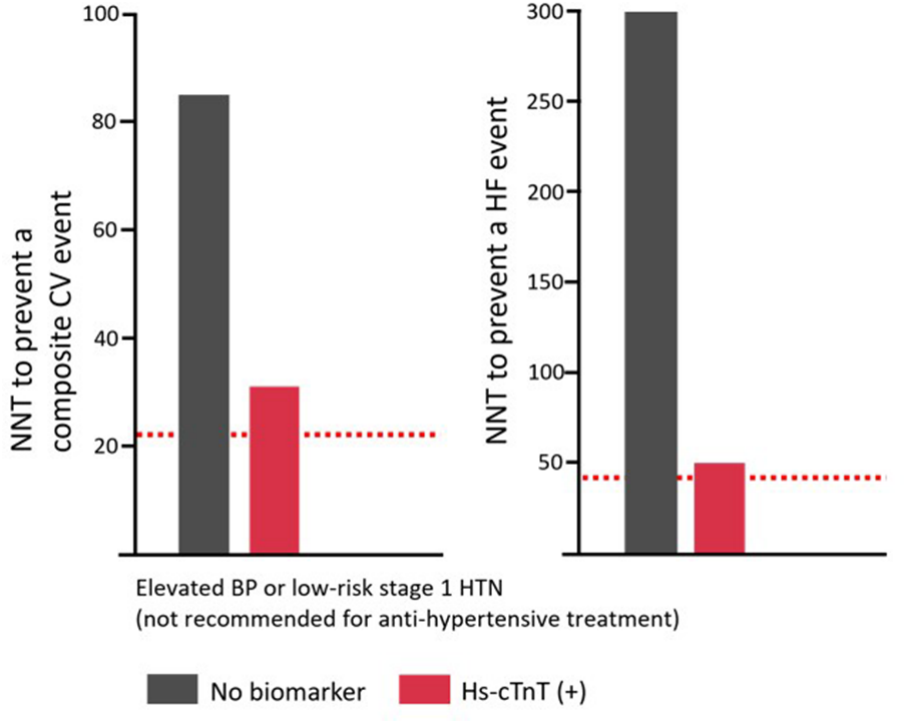
The 10-year number needed to treat to prevent an incident composite CV event, or HF (right), across 2017 ACC/AHA BP guideline–recommended treatment groups stratified by hs-cTnT [Adapted from Pandey et al. (12)]. The dotted red line represents the 10-year NNT to prevent an incident composite CV event (left) and HF (right) for the group with stage 2 HTN (BP≥160/100 mmHg). Definitions: Elevated BP, 120–129/<80 mm Hg; stage 1 HTN, 130–139/80 to 89 mm Hg; stage 1 high-risk HTN was defined by the presence of any of the following: PCE-estimated 10-year ASCVD risk ≥10%, diabetes mellitus, estimated GFR <60 ml/min/1.73 m^2^, or age ≥65 years with systolic BP ≥130 mmHg; in the absence of all of these risk factors, individuals with stage 1 HTN were classified as low risk. An hs-cTnT≥6 ng/L is considered elevated. NNT, number needed to treat; CV, cardiovascular; HF, heart failure; BP, blood pressure; HTN, hypertension; hs-cTnT, high-sensitivity cardiac troponin T; ASCVD, atherosclerotic cardiovascular disease; GFR, glomerular filtration rate; PCE, pooled cohort equation. Adapted with permission from “Prevalence of elevated hs-cTnT or NT-proBNP across 2017 ACC/AHA BP guideline recommended treatment groups” by Berry et al. (13). The Creative Commons license does not apply to this content. Use of the material in any format is prohibited without written permission from the publisher, Wolters Kluwer Health, Inc. Please contact permissions@lww.com for further information.

The strategy of incorporating hs-cTn assays into CV risk stratification to guide therapeutic decision-making has been tested retrospectively in existing community cohorts with well-defined centrally adjudicated outcomes. In an analysis inclusive of three United States-based cohorts (Atherosclerosis Risk in Communities Study, Dallas Heart Study, and Multiethnic Study of Atherosclerosis), Pandey et al. examined individuals without known CVD, including coronary heart disease, stroke, and HF, who were also not on antihypertensive medication at the time of enrollment (12). The investigators found that the incorporation of cardiac biomarkers [hs-cTnT or amino terminal B-type natriuretic peptide (NT-proBNP)] in risk assessment algorithms enhanced the risk stratification of patients, identifying individuals who could potentially benefit from antihypertensive treatment. This was particularly true for participants with elevated blood pressure (BP) in addition to those with low-risk stage 1 hypertension who are not typically recommended antihypertensive medications by current guidelines. As shown in Figure 2, including hs-cTnT levels stratified both categories of patients, resulting in large differences in the number needed to treat for either a composite CV outcome or incident HF. These findings suggest that a knowledge of hs-cTn levels could potentially prompt early initiation of pharmacologic BP treatment, thereby reducing incident symptomatic CV events for these frequently encountered ambulatory patients. Furthermore, the fact that the associated risk linked to elevated hs-cTnT levels is potentially modifiable through pharmaceutical intervention lends clinical significance to these findings. Similar findings were noted for NT-proBNP measurements (13).

While many studies have investigated the role of mild hs-cTn elevations in the general population, similar efforts have been made in higher-risk ambulatory populations with known ASCVD. Marston et al. explored the potential benefit of incorporating hs-cTnI levels for risk evaluation within the framework of the 2018 AHA/ACC cholesterol management guidelines (14). In their exploratory analysis of patients with prior MI from the Patients With Prior MI (PEGASUS-TIMI 54) trial, participants were classified as either “very high-risk” or “lower-risk” ASCVD based on the 2018 AHA/ACC cholesterol management guidelines. Incorporating a baseline hs-cTnI level (measured with the Abbott Architect hs-cTnI assay) further stratified risk within these categories. For example, patients classified as “very high-risk” overall had a 3-year event rate (CV death, MI, and stroke) of 8.8% compared with 5.0% for the lower-risk patients. However, when clinically determined “very high-risk” patients were further assessed based on hs-cTnI levels, those with hs-cTnI<2 ng/L had an event rate of only 2.7%, while those with hs-cTnI>6 ng/L had a more than fivefold higher event rate of 14.3%. Overall, approximately 12% of the participants in the study would have their risk reclassified with the addition of the hs-cTnI assay, potentially affecting their secondary prevention treatments and long-term disease outcomes (14).

These findings indicate that using hs-cTn levels for risk stratification and blood pressure management is promising, both for the general population and for secondary prevention in individuals with known ASCVD. While this offers an opportunity for personalized medicine, validation with prospective clinical trials is needed.

Risk factors such as cholesterol levels and blood pressure are compelling biomarkers because they are well-established components of the causal pathways underlying symptomatic CVD and death. It is less likely that ongoing myocyte cell death reflected by circulating hs-cTns levels is directly involved in the causal pathway for CVD. Instead, it appears to be a subclinical organ-specific consequence but remains an important marker of disease.

### 2.2 Correlation of hs-cTn with cardiac imaging

An important question is how well early biochemical elevations of myocyte injury reflected by hs-cTns levels correspond with early indicators of myocyte damage as detected through cardiac imaging techniques. This association was estimated in the MESA (Multi-Ethnic Study of Atherosclerosis) cohort in which baseline elevations in hs-cTnT assays among individuals without overt CVD were associated with the presence of replacement fibrosis, which was evidenced by late gadolinium enhancement (LGE) in cardiac magnetic resonance (CMR). Interestingly, there was no association of the biomarker level with an ischemic pattern seen with MI, suggesting a non-ischemic etiology for measurable circulating hs-cTnT levels in these CVD-free individuals. These elevations also correlated with an increased likelihood of longitudinal changes in the left ventricle (LV) remodeling represented by an increase in LV mass and end-diastolic volume as assessed by CMR. Notably, however, there was no correlation with hs-cTnT level in the MESA with a subsequent reduction in systolic function, as reflected by left ventricular ejection fraction (LVEF) (15). Increased interstitial fibrosis was also associated with increased hs-cTnT levels in the same cohort (16). These associations are of interest when planning future studies for evaluating the performance of hs-cTns as a screening test in the general population. However, hs-cTns levels are not static and frequently change over time. Consequently, they can reflect the progression of subclinical disease and potentially the efficacy of therapies designed to prevent symptomatic CVD and death. In the following section, we explore the current evidence regarding longitudinal change in hs-cTn levels, which can enhance both screening and measurement of treatment efficacy.

### 2.3 Longitudinal measures of cardiac troponin for screening, prognosis, and assessing treatment efficacy

The measurement of longitudinal changes in hs-cTn levels in asymptomatic individuals both with and without known CVD is intriguing because rising levels intuitively suggest the acceleration of subclinical disease. Indeed, there have been consistent findings across multiple general population cohorts involving people without known CVD that demonstrate that a 25%–50% increase in hs-cTns levels over the course of 1–6 years is associated with a poor prognosis. These rising hs-cTn levels correlate with a higher risk of incident ASCVD, HF, and CV death (17–19). This longitudinal finding can also be noted in patients with known CVD. In the Bypass Angioplasty Revascularization Investigation in Type 2 Diabetes trial (BARI-2D), an increase of ≥25% over 1 year was associated with an increased risk of CV death, MI, stroke, or HF compared with those with a <25% rise (20).

In the Long-Term Intervention with Pravastatin in Ischemic Disease (LIPID) study, increases in hs-cTnI levels over the first year were predictors of CV death, MI, and all-cause death. This provided independent prognostic information in patients with stable CAD beyond what a single baseline measurement alone could offer, suggesting that serial monitoring of cardiac troponin levels can reflect dynamic changes in cardiovascular risk (21). However, while pravastatin treatment resulted in a slightly greater reduction in hs-cTnI levels than placebo, this change in level did not account for any of the effects of pravastatin in reducing the number of CV events. There is little evidence that longitudinal changes in hs-cTn levels in ambulatory adults are the result of ischemic events or “silent MIs”; therefore, it is perhaps not surprising that hs-cTn change did not account for the efficacy of pravastatin as the biomarker is not likely to act as an intermediary between treatment and clinical outcomes. Therefore, as of now, hs-cTn does not have a role in measuring the efficacy of drug therapy to reduce coronary heart disease events.

Lastly, interpretation of longitudinal changes as a result of preventive treatments remains a work in progress. For example, while examining the effects of intensive blood pressure control on hs-cTnT levels in the Systolic Blood Pressure Intervention Trial (SPRINT), investigators found that although increased hs-cTnT levels correlated with higher risks for incident HF and death, intensive systolic BP lowering led to an unexpected rise in hs-cTnT levels. This finding was primarily attributed to a simultaneous decline in the estimated glomerular filtration rate and prompts further questions about the interchangeability of hs-cTnT and I for screening in this population (13).

### 2.4 Are hs-cTnT and hs-cTnI interchangeable?

Hs-cTnT and I are generally thought to be interchangeable with respect to the diagnosis of MI, with a high correlation between their respective levels (22). However, in ambulatory adults, the correlation between the two biomarkers is only moderate (23, 24). Multiple factors, including biologic and analytical differences in a chronic setting, may influence the presence of this moderate correlation. From a biologic standpoint, it is interesting to note that hs-cTnT has also been found to be elevated in patients with neuromuscular diseases and skeletal muscle disorders, whereas hs-cTnI is not elevated in these individuals (25, 26). With respect to an analytical difference, while there is only one commercial hs-cTnT assay, there are multiple hs-cTnI assays available for clinical use (27). Each hs-cTnI assay targets different epitopes and thus may have different affinities to the epitopes and susceptibilities to endogenous cTn antibodies that may influence these assays (28). How these differences may influence the prediction of future CVD events based on low-level elevations is uncertain. Some studies have suggested that cTnT and I are not interchangeable for risk prediction, particularly with respect to non-fatal ASCVD events. Welsh et al. demonstrated in a Scottish community-based cohort of over 19,500 individuals that hs-cTnI and hs-cTnT showed similarity for incident CVD death and HF prediction, but only cTnI predicted incident MI (24). However, coronary imaging studies have demonstrated an association between hs-cTnT and extent and the “vulnerability” of coronary plaque (29, 30). Two meta-analyses have addressed separate CVD endpoints for hs-cTnI and hs-cTnT in ambulatory populations (8, 31). Both noted that despite the high heterogeneity between individual studies, both cTnT and I levels were predictive for all of the CVD endpoints.

## 3 Future directions and ongoing discussions

The current improved analytical hs-cTn assays detect cTnT and cTnI levels in most asymptomatic adults, seemingly reflecting ongoing baseline myocyte cell death. The addition of these inexpensive, ubiquitously available clinical blood tests that measure myocyte injury is potentially promising to augment the screening of hypertensive patients such that additional patients can be identified for antihypertensive treatment targeting the lower thresholds reported in the SPRINT. Ensuing treatment initiatives may substantially reduce the risk of new-onset HF and other CV events in the general population of the United States. Screening based on the data presented above from the three United States-based cohorts is intriguing, but several questions remain from that exploratory analysis. It is crucial to recognize that we still need to confirm the cost-effectiveness and precision of this method in prospective studies designed with this intent. Equally significant is the challenge of determining optimal management strategies for individuals identified as higher risk through positive screening results. Tailoring interventions based on biomarker results demands thoughtfulness to avoid unnecessary medical interventions or overlooking individuals who might benefit from proactive care. Moreover, the overarching question is whether subjecting a higher-risk group identified by hs-cTn levels to more aggressive management and intensified monitoring would translate to tangible long-term improvements in their clinical outcomes. Conversely, we must consider whether detection of subclinical disease with hs-cTn may simply result in early detection, subject a patient to the side effects of treatment, but ultimately not extend longevity beyond what would have occurred without screening. There is a compelling case for a pragmatic, randomized clinical trial to implement antihypertensive treatments in the early stages of hypertension in patients with higher hs-cTn levels. While early identification of risk is essential, understanding whether intensified interventions truly alter the trajectory of the disease remains a critical piece of the puzzle.
